# Lipedema in Women and Its Interrelationship with Endometriosis and Other Gynecologic Diseases: A Scoping Review

**DOI:** 10.3390/biomedicines14010122

**Published:** 2026-01-07

**Authors:** Diogo Pinto da Costa Viana, Adriana Luckow Invitti, Eduardo Schor

**Affiliations:** 1 Department of Gynecology, Escola Paulista de Medicina, Federal University of Sao Paulo (EPM-UNIFESP), Sao Paulo 04024-002, Brazil; 2 Brazilian Society for Research and Teaching in Medicine (SOBRAPEM), Sao Paulo 01318-901, Brazil; 3 Brazilian Society of Obesity Medicine (SBEMO), Florianópolis 88070-800, Brazil

**Keywords:** lipedema, women, endometriosis, genital diseases, female, polycystic ovary syndrome (PCOS), uterine fibroids (leiomyoma)

## Abstract

**Background**: Emerging evidence suggests that lipedema may share hormonal, inflammatory, and genetic mechanisms with gynecologic diseases, particularly endometriosis. However, the extent and nature of these interrelationships remain poorly characterized, supporting the need for this scoping review. **Objectives**: To map and synthesize the available evidence on the clinical, pathophysiological, and epidemiological interrelationships between lipedema in women, endometriosis, and other gynecologic diseases. **Methods**: Searches were conducted in international and regional health databases, including MEDLINE (PubMed), CINAHL, Scopus, Embase, Web of Science, the Cochrane Library, LILACS/VHL, APA PsycInfo, SciELO, Epistemonikos, and La Referencia, as well as grey literature sources and relevant institutional websites. There were no language restrictions. The search period began in 1940, the year in which lipedema was first described by Allen and Hines. Study selection followed a two-stage process conducted independently by two reviewers, consisting of title and abstract screening followed by full-text review. Data extraction was performed using a pre-developed and peer-reviewed instrument covering participants, concept, context, study methods, and main findings. The review protocol was registered in the Open Science Framework. **Results**: Twenty-five studies from ten countries were included. Synthesized evidence supports the characterization of lipedema as a systemic condition with metabolic and hormonal dimensions. Key findings include symptom onset linked to reproductive milestones, a high frequency of gynecologic and endocrine comorbidities, and molecular features overlapping with steroid-dependent pathologies. These patterns reflect a recent shift from a predominantly lymphovascular paradigm toward a more integrated endocrinometabolic framework. **Conclusions**: The findings indicate that lipedema clusters with hormone-sensitive gynecologic and endocrine features across reproductive life stages.

## 1. Introduction

Lipedema is a chronic, progressive adipose tissue disorder that predominantly affects women and remains widely underrecognized in clinical practice [1,2]. It is characterized by a symmetrical and disproportionate accumulation of subcutaneous fat in the extremities, typically sparing the hands and feet, and is frequently associated with pain, easy bruising, and resistance to weight-loss interventions. Since its initial description by Allen and Hines in 1940, lipedema has often been misclassified as simple obesity, lymphedema, or venous disease, contributing to substantial diagnostic delay and fragmented care pathways [3].

Beyond its characteristic adipose phenotype, lipedema exhibits a striking temporal sensitivity to female reproductive life stages. Symptom onset or progression is commonly reported during puberty, pregnancy, and the menopausal transition, suggesting that hormonal fluctuations may act as modulators of disease expression rather than primary etiologic drivers. This observation has prompted increasing interest in potential interrelationships between lipedema and gynecologic conditions that share hormone-responsive and inflammatory features, particularly endometriosis.

Endometriosis is a chronic, estrogen-dependent inflammatory disorder affecting approximately 10% of women of reproductive age [4] and represents a leading cause of pelvic pain, dysmenorrhea, dyspareunia, and infertility [5]. Given the distinct hormonal chronodependence of lipedema, a critical question arises regarding its potential intersection with other estrogen-dependent pathologies. This scoping review was conducted not to assert a confirmed comorbidity, but to investigate whether the substantial burden of pelvic symptoms reported in clinical practice [6] correlates with diagnosed gynecological disease. By mapping these intersections, we aim to distinguish between confirmed diagnoses (such as endometriosis) and functional symptom clusters, addressing a potential gap in women’s health screening.

Despite growing interest in these potential associations, the existing literature remains fragmented. Individual studies and narrative reviews have proposed overlapping clinical patterns and biological mechanisms, yet no prior scoping or systematic review has comprehensively mapped the extent, nature, and characteristics of evidence addressing the interrelationship between lipedema and gynecologic diseases. Preliminary searches of MEDLINE, JBI Evidence Synthesis, and the Cochrane Database confirm the absence of a dedicated evidence synthesis focused on this topic.

Given the heterogeneity of study designs, populations, and outcomes, a scoping review represents the most appropriate methodological approach to clarify conceptual boundaries, identify knowledge gaps, and synthesize the available evidence without inferring causality or disease equivalence. Accordingly, this scoping review aims to systematically map the clinical, pathophysiological, and epidemiological evidence on the interrelationships between lipedema in women and gynecologic disorders, with a primary focus on endometriosis, while also considering other hormonally responsive gynecologic conditions where relevant.

## 2. Methods

### 2.1. Study Design and Protocol Registration

This scoping review was conducted in accordance with the JBI methodology for scoping reviews [7] and is reported following the Preferred Reporting Items for Systematic Reviews and Meta-Analyses extension for Scoping Reviews (PRISMA-ScR) guidelines [8]. To ensure transparency, reproducibility, and to reduce the risk of selective outcome reporting, the review protocol was developed a priori and registered in the Open Science Framework (OSF) under the identifier DOI: 10.17605/OSF.IO/D65GS (https://osf.io/d65gs/overview) (accessed on 2 September 2025) [9].

### 2.2. Review Question

What are the clinical, pathophysiological, and epidemiological interrelationships between lipedema in women and endometriosis and other gynecologic diseases?

### 2.3. Eligibility Criteria

#### 2.3.1. Participants

This review included women of any age with a clinical diagnosis of lipedema, as originally defined by Allen and Hines (1940, 1951) [3,10] as a painful and symmetric disorder characterized by disproportionate fat distribution in the extremities relative to the trunk. The diagnosis may have been established based on clinical criteria, medical records, or assessments reported in the primary studies. Women with lipedema at any clinical stage were considered eligible, irrespective of the presence of obesity, vascular comorbidities such as varicose veins, or other associated conditions. Studies addressing physical, metabolic, functional, psychosocial, or quality-of-life aspects related to lipedema were eligible for inclusion.

#### 2.3.2. Concept

The review aimed to map the available evidence on the clinical, pathophysiological, and epidemiological interrelationships between lipedema in women and gynecologic diseases, with a particular focus on endometriosis. Endometriosis was considered as a reference gynecologic condition given its established inflammatory and estrogen-dependent biology, characterized by the growth of endometrium-like tissue outside the uterine cavity and associated with pelvic pain, infertility, and impaired quality of life.

In addition to endometriosis, other gynecologic conditions potentially associated with lipedema were considered, including polycystic ovary syndrome (PCOS), uterine fibroids and premenstrual dysphoric disorder (PMDD).

The review sought to identify shared clinical patterns, commonly reported pathophysiological mechanisms such as hormonal alterations, chronic inflammation, insulin resistance, or lymphatic dysfunction, and reported epidemiological associations among these conditions. This broad conceptual definition allowed inclusion of observational, clinical, pathophysiological, and genetic studies contributing to understanding potential interrelationships between lipedema and gynecologic diseases. Additional gynecologic conditions were considered if identified through the search strategy and met the conceptual criteria.

Studies focusing exclusively on obesity, lymphedema, or other metabolic or endocrine disorders without clear distinction from lipedema were excluded. Reports addressing gynecologic diseases without explicit reference to lipedema or lacking standardized diagnostic definitions of the included conditions were also excluded.

#### 2.3.3. Context

The context of this review encompassed all women-centered health care settings, including primary, secondary, and tertiary care, as well as specialized services in gynecology, reproductive health, endocrinology, angiology and vascular medicine, nutrition, physiotherapy, mental health, and surgery. Multidisciplinary and integrative care settings involving clinical, surgical, psychological, and functional rehabilitation approaches were included. Studies conducted across diverse geographic and cultural regions were considered, acknowledging that sociocultural and structural factors may influence diagnosis, access to care, and management of both lipedema and gynecologic diseases. Studies conducted outside formal health care settings, such as social media reports or purely personal narratives without clinical evaluation, and studies lacking clear description of the health care context were excluded.

### 2.4. Information Sources and Search Strategy

This scoping review considered analytical observational study designs, including prospective and retrospective cohort studies, case–control studies, and analytical cross-sectional studies, as these designs are appropriate for examining clinical, pathophysiological, and epidemiological associations. Descriptive observational designs, such as case series, individual case reports, and descriptive cross-sectional studies, were also included, recognizing their potential value in an underexplored field such as lipedema.

Systematic reviews and other types that met the inclusion criteria were included insofar as they contributed to synthesis and organization of evidence on interrelationships among the conditions of interest. Reports, guidelines, working papers, white papers, and relevant websites were identified through database and gray literature searches and screened according to the eligibility criteria. Reference lists of included reviews were hand searched for additional relevant studies. Opinion pieces and articles lacking an empirical basis were excluded.

A three-step search strategy was applied. First, an initial limited search was conducted in MEDLINE via PubMed, JBI Evidence Synthesis, the Cochrane Library, OSF, and PROSPERO to identify relevant articles. Second, keywords and indexing terms from relevant titles and abstracts were used to develop a comprehensive search strategy using controlled vocabularies, including DeCS/VHL, MeSH, and Emtree. Third, the finalized search strategy was adapted for each database and information source searched, including the LilacsPlus Collection of the Virtual Health Library, MEDLINE via PubMed, CINAHL, Academic Search Premier, Food Science Source, FSTA, SocINDEX, SPORTDiscus, Scopus, Embase, Web of Science, APA PsycInfo, SciELO, Epistemonikos, La Referencia, and the Cochrane Library. Gray literature sources included Science.gov, OpenGrey, arXiv, Google Scholar, and websites of relevant professional organizations, including the Lipedema World Alliance and the International Lipoedema Association. Documents from governmental and regulatory agencies were also consulted.

Studies published in any language were eligible. The search period began in 1940, corresponding to the original clinical description of lipedema by Allen and Hines. The search strategy was reviewed by a reviewer trained in the JBI methodology, using the Peer Review of Electronic Search Strategies (PRESS) guideline. The search strategy relied primarily on lipedema-related terms without the use of restrictive gynecological ‘AND’ operators. This broad, participant-centered approach was a deliberate scoping decision designed to maximize sensitivity. By retrieving the entire corpus of lipedema literature and screening for gynecological outcomes at the full-text level, we ensured that relevant studies describing pelvic symptoms (e.g., ‘cycle-dependent pain’)—which might not be indexed with specific gynecological keywords in databases—were not overlooked. The final search was executed on 16 September 2025. The complete search strategy is presented in Table 1.

After the search was completed, all identified citations were exported to EndNote version 21 (Clarivate Analytics, Pennsylvania, PA, USA) for reference management and duplicate removal. The deduplicated records were then imported into Rayyan (Qatar Computing Research Institute, Doha, Qatar) [11], a web-based application developed to assist in screening and selecting studies in evidence syntheses. Prior to formal screening, a pilot test was conducted to ensure consistent application of eligibility criteria.

Titles and abstracts were independently screened by two reviewers according to predefined inclusion and exclusion criteria. Sources considered potentially relevant were retrieved in full text and independently assessed for eligibility. Reasons for exclusion at the full-text stage were documented and are reported in the final scoping review.

Any disagreements between reviewers at any stage of the selection process were resolved through discussion. When consensus could not be reached, a third reviewer was consulted to arbitrate eligibility. The overall research results and the study selection process are reported according to PRISMA-ScR recommendations and presented in a PRISMA flowchart.

The reference lists of all included evidence sources were examined to identify additional studies.

### 2.5. Data Charting and Synthesis

A standardized data charting instrument was developed in Microsoft Excel specifically for this scoping review to ensure consistent and comprehensive extraction of relevant information across studies. The instrument captured data related to study identification and methodological characteristics, including authorship, year of publication, title, country or study setting, source journal, study objectives, study design, sample size, target population, age range, and reported inclusion and exclusion criteria.

Detailed information regarding the conditions investigated was also extracted, including diagnostic criteria or methods used to identify lipedema, the gynecologic conditions assessed, and the diagnostic approaches applied for gynecologic evaluation. In addition, the instrument recorded the types of interrelationships examined across clinical, pathophysiological, and epidemiological domains, as well as reported outcomes, key findings, and relevant quantitative or qualitative measures. Data related to care context, psychosocial dimensions, and sociocultural factors were extracted to support appropriate contextualization of the evidence.

Extracted data were synthesized descriptively and presented as a narrative summary, complemented by tables, figures, and conceptual maps where appropriate. The format of presentation was adapted according to the availability, consistency, and quality of the reported information, and any deviations from the planned synthesis approach are documented in the final review.

The accompanying narrative synthesis describes participant characteristics, the main outcomes reported in the included studies, with emphasis on the clinical, pathophysiological, and epidemiological interrelationships between lipedema, endometriosis, and other gynecologic diseases.

## 3. Results

**Figure 1:**
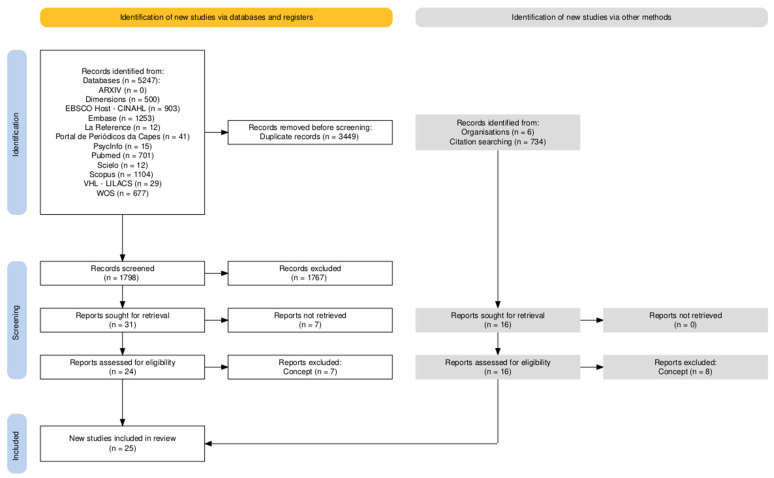
PRISMA flowchart for identifying, selecting, and including articles.

The initial search in the database identified 5247 records. After removing 3449 duplicates, 1798 records were analyzed, of which 1767 were excluded. Thirty-one studies were selected for full-text retrieval; however, seven could not be retrieved. Of the 24 reports assessed for eligibility in the databases, seven were excluded for not meeting the conceptual eligibility criteria. In parallel, 16 records identified by other methods were assessed for eligibility, with eight excluded based on the concept. At the end of the selection process, 25 studies were included in the review (Figure 1) [12].

The included studies were published between 2010 and 2025, with a progressive increase in scientific production observed in recent years. The temporal distribution was as follows: 2010 (*n* = 1) [1], 2014 (*n* = 1) [13], 2015 (*n* = 1) [14], 2019 (*n* = 3) [15,16,17], 2020 (*n* = 2) [18,19], 2021 (*n* = 1) [20], 2022 (*n* = 2) [21,22], 2023 (*n* = 3) [23,24,25], 2024 (*n* = 6) [26,27,28,29,30], and 2025 (*n* = 6) [31,32,33,34,35,36]. A clear concentration of publications is observed from 2023 onwards, with 15 studies (58%) published in the last three years.

### 3.1. Characteristics of Included Studies

#### 3.1.1. Geographical Distribution

**Figure 2:**
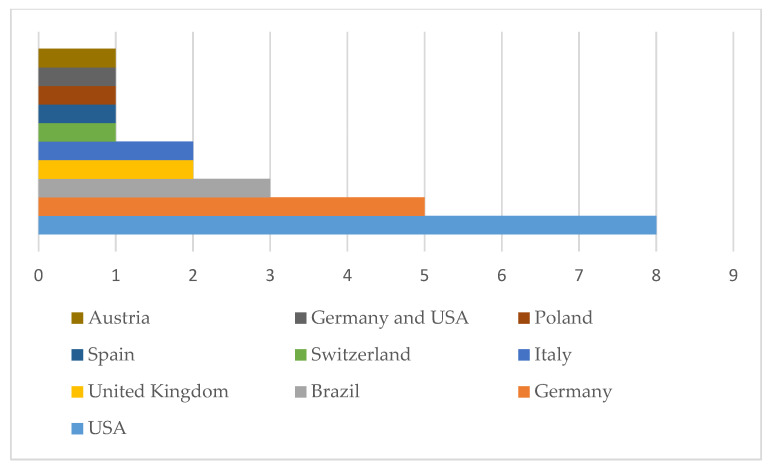
Distribution of studies by country.

The geographical distribution of the included studies and the predominant research focus across countries are summarized in Figure 2. Overall, the studies were conducted across multiple countries, with a predominance of the United States—USA (*n* = 8, 32%), followed by Germany (*n* = 5, 20%) and Brazil (*n* = 3, 12%). Other represented countries included the United Kingdom (*n* = 2), Italy (*n* = 2), Austria (*n* = 1), Poland (*n* = 1), Spain (*n* = 1), and Switzerland (*n* = 1). One study was conducted through a collaborative effort between Germany and the United States—USA (Figure 2).

#### 3.1.2. Study Designs

The included studies demonstrated substantial methodological heterogeneity, reflecting the exploratory and multidisciplinary nature of research on lipedema. Narrative literature reviews were the most frequently identified design, accounting for seven publications (28%). Cross-sectional observational studies, including online survey-based designs, comprised four studies (16%). Observational studies of other types accounted for six publications (24%), encompassing retrospective cohort analyses, multi-omics investigations, and medical record reviews.

Additional study designs were less frequent and included a methodological optimization study (*n* = 1), a case report presented as a poster abstract (*n* = 1), in vitro experimental studies (*n* = 1), multilevel experimental analyses (*n* = 1), observational genetic studies employing whole-exome sequencing combined with molecular modeling approaches (*n* = 1), theoretical models and perspective articles (*n* = 1), and cohort study reviews (*n* = 1). Overall, this diversity of study designs underscores the early-stage, hypothesis-generating character of the field and the absence of a dominant methodological paradigm.

#### 3.1.3. Sample Characteristics

The largest studies included a retrospective analysis of 1803 patients with a clinical diagnosis of lipedema, a cross-sectional study of 860 participants with lipedema who completed online questionnaires, and an observational study involving 381 women affected by the condition.

Medium-sized studies investigated samples ranging from 50 to 360 participants. Smaller studies, typically characterized by more specific experimental or genetic designs, included samples of 136 women with lipedema and 49 controls for multi-omics analyses, with subgroups of 14 patients and 7 controls for transcriptomic analyses; 51 patients (50 women and 1 man); 32 women diagnosed with lipedema and 14 matched controls; 20 adult women (10 with stage II lipedema and 10 healthy controls); 12 family members (3 affected and 9 unaffected); and 9 participants (4 healthy women and 5 patients with lipedema). One case report described a single 50-year-old patient in amenorrhea with manifestations of lipedema.

#### 3.1.4. Target Population

The predominant population across studies consisted of adult women with a clinical or physician-confirmed diagnosis of lipedema. Most studies focused specifically on women with primary, non-syndromic lipedema, often across different disease stages (stages 1–3, with an emphasis on stage II). Some studies included control groups matched for age and body mass index, composed of healthy women without adipose tissue disorders. Experimental studies investigated human female subcutaneous adipose tissue. Notably, although lipedema predominantly affects women, representing 99.2% to 100% of samples in most studies, two studies reported the inclusion of men: one study of 51 patients included one man, and another large initial cohort of 1846 patients excluded two men from the final analysis. Some studies focused on specific populations, such as non-obese women with early-stage lipedema, patients undergoing tumescent liposuction, or women with lipedema and estrogen-dependent gynecological disorders. The age range investigated predominantly spanned adults aged 19 to 70 years, with one study including patients aged 17 years or older.

### 3.2. Study Characteristics and the Global Research Shift

An analysis of the included literature reveals a clear geographical and conceptual shift in lipedema research over the last decade. Historically, the majority of published studies originated from Central Europe, particularly Germany and Austria, where lipedema was predominantly framed within a lymphological context, with emphasis on edema, venous or lymphatic alterations, and surgical outcomes. In contrast, studies published from approximately 2019 onward increasingly incorporated perspectives from endocrinology, genetics, and molecular biology. This more recent body of work has been driven primarily by research groups based in the United States, Italy, and Brazil, with growing attention to steroidogenic enzymes, intracellular hormone metabolism, and genetic susceptibility markers [18,21,37].

In parallel, large clinical cohorts from Spain, Switzerland, and Germany have contributed detailed epidemiological descriptions of lipedema and associated comorbidities. The Spanish cohort reported by Simarro et al. (2025) [36] represents the largest dataset to date (*n* = 1803), providing comprehensive characterization of clinical features in a Mediterranean population. Additional cohorts from Switzerland (*n* = 381) and Italy (*n* = 360) offer corroborative data across distinct European settings [28,30]. Across these cohorts, symptom onset was most commonly reported during early adolescence, between 12 and 16 years of age, whereas formal diagnosis typically occurred in the fourth or fifth decade of life, indicating a diagnostic delay exceeding two decades.

Notably, several cohorts reported discordance between lower extremity adiposity and systemic metabolic markers. Many participants exhibited normal glycemic and lipid profiles despite elevated body mass index and marked lower body fat accumulation. These observations distinguish lipedema from common obesity phenotypes and have contributed to increasing discussion of lipedema as a condition characterized by hormonally responsive and metabolically distinct adipose tissue. While these findings challenge a purely lymphovascular conceptualization, they should be interpreted descriptively and underscore the need for integrative models rather than replacement of existing frameworks.

**Figure 3:**
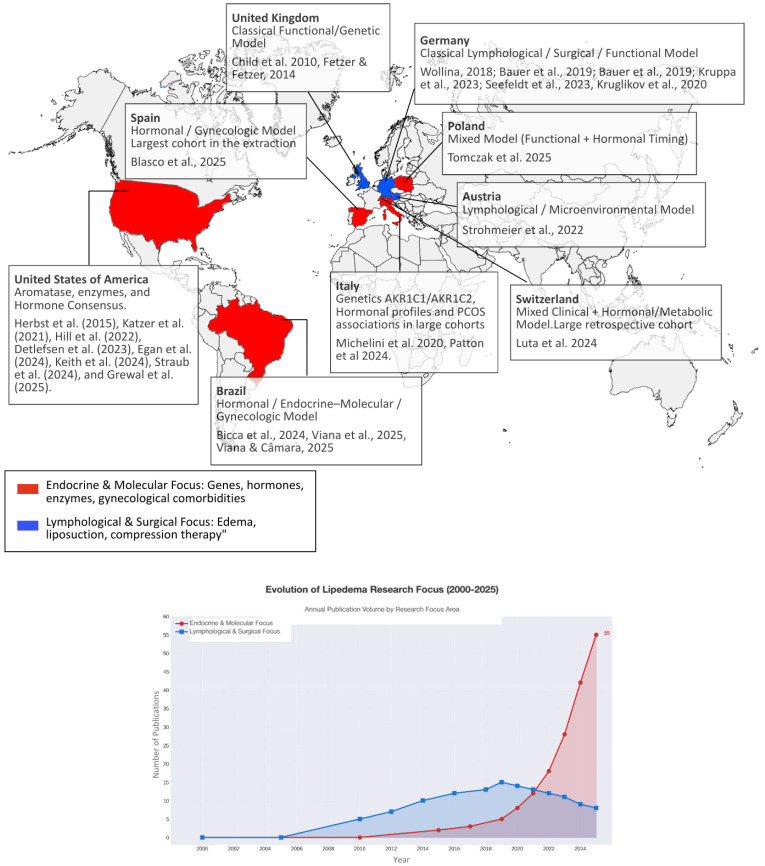
Geographic distribution and evolution of lipedema research focus. World map summarizing the geographic distribution of lipedema research and the predominant thematic focus across countries, distinguishing endocrine, molecular, and gynecologic approaches from lymphological and surgical approaches. Color intensity reflects publication volume; icons indicate predominant research focus. The lower panel illustrates the temporal evolution of publication volume by research focus area between 2000 and 2025. Legend: AKR1C1—Aldo-keto reductase family 1 member C1, KR1C2—Aldo-keto reductase family 1 member C2, PCOS—Polycystic ovary syndrome [1,13,14,15,16,17,18,19,20,21,22,23,24,25,26,27,28,29,30,31,32,33,34,35,36].

This distribution indicates a predominance of research conducted in high-income countries, with contributions mainly from Europe, North America, and Latin America (Figure 3).

### 3.3. Incidence of Gynecological and Endocrine Comorbidities

**Figure 4:**
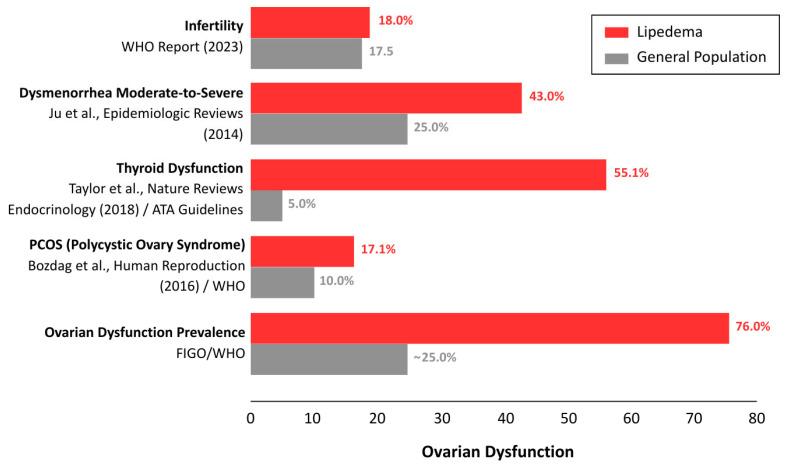
In the largest observational cohort analyzed to date, involving 1803 women in Spain, a clinically defined category termed inflammatory ovarian dysfunction was reported in 76.0% of participants [36]. This designation encompassed a spectrum of ovulatory and menstrual disturbances, including irregular cycles, heavy menstrual bleeding, and pelvic pain, and was derived from clinical history and medical record documentation rather than standardized reassessment using uniform diagnostic criteria. As this terminology reflects the classification adopted by the primary study rather than a universally established diagnostic entity, comparisons across cohorts should be interpreted with caution. Nevertheless, the high frequency of these features raises the possibility of interacting biological pathways linking adipose tissue dysfunction with ovarian and inflammatory processes, without implying causality [38,39,40,41,42].

Synthesized data from the included cohorts indicate that gynecological and endocrine conditions are frequently reported among individuals with lipedema, with higher reported prevalence of pelvic pain–related symptoms and selected endocrine comorbidities compared with population-based estimates (Figure 4). Population-based reference estimates were derived from established epidemiological sources, including the World Health Organization (WHO, 2023) [38], Ju et al. (2014) [39], Taylor et al. (2018) [40], Bozdag et al. (2016) [41], and reports from the International Federation of Gynecology and Obstetrics (FIGO) [42]. These findings are derived primarily from observational cohorts and should be interpreted as descriptive patterns rather than measures of relative risk or causality. Furthermore, comparisons with general population estimates must be interpreted with caution due to inherent selection bias. The lipedema cohorts were largely derived from specialized tertiary referral centers, where patients likely present with greater disease severity and comorbidity burden than the general lipedema population.

Furthermore, comparisons with general population estimates must be interpreted with caution due to inherent selection bias. The lipedema cohorts were largely derived from specialized tertiary referral centers, where patients likely present with greater disease severity and comorbidity burden than the general lipedema population. To address this heterogeneity and clarify diagnostic certainty, Table 2 stratifies these gynecological findings by method of ascertainment, explicitly distinguishing between surgically or medically confirmed diagnoses and symptom-based reports.

In the largest observational cohort analyzed to date, involving 1803 women in Spain, 76.0% of participants presented with ‘Ovulatory Dysfunction and Menstrual Disorders’ (originally classified by the authors as ‘inflammatory ovarian dysfunction’) [36]. It is important to explicitly clarify that this term does not represent a standardized universal diagnostic entity found in gynecological classification systems (e.g., FIGO). Rather, it was employed by the primary study authors as a clinical symptom cluster encompassing a spectrum of ovulatory and menstrual disturbances, including irregular cycles, heavy menstrual bleeding, and pelvic pain, derived from clinical history rather than standardized prospective monitoring.

To enhance interpretability across heterogeneous gynecological outcomes reported in the included studies, a descriptive clustering approach was applied during data synthesis. No statistical cluster analysis or meta-analytic pooling was performed. Instead, conceptually related outcomes were grouped into shared clinical domains, including pelvic pain–related manifestations (e.g., moderate to severe dysmenorrhea and cycle-related pelvic pain), ovarian dysfunction, menstrual disturbances, and endocrine comorbidities. Incidence estimates for these domains were obtained through direct extraction of numerical and data from primary study tables, with weighted proportions calculated using the number of women assessed for each specific outcome as the denominator. This approach allowed integration of partially overlapping outcomes while avoiding artificial precision and minimizing double counting.

Using this framework, a pelvic pain–related symptom cluster was consistently observed. In the German cohort (*n* = 860), 43.0% of women reported significant menstrual complaints, including moderate to severe dysmenorrhea and cycle-related worsening of lower extremity pain [25], while 32.5% of participants in the Italian cohort reported chronic menstrual irregularities [28]. Importantly, the term pelvic pain cluster is used descriptively to denote recurrent co-reporting of symptoms across cohorts and does not represent a statistical clustering analysis. These symptom frequencies contrasted with the comparatively low prevalence of surgically confirmed endometriosis (4.2%), suggesting potential underrecognition or alternative attribution of pelvic symptoms in routine clinical practice. Structural gynecological findings, including leiomyomas (15.2%) and ovarian cysts (23.5%), were also reported, consistent with patterns observed in estrogen-responsive tissues.

Similarly, the reported prevalence of Polycystic Ovary Syndrome (PCOS) ranged from 12.6% to 17.1% across weighted analyses of Italian and German cohorts, exceeding commonly cited population-based estimates of approximately 8–10% [28]. These figures reflect previously established clinical diagnoses rather than standardized reassessment and have led some authors to propose overlapping metabolic features, such as insulin resistance and hyperandrogenism, that may influence adipose tissue behavior in both conditions, although these hypotheses remain untested.

Beyond reproductive disorders, thyroid dysfunction, predominantly autoimmune thyroiditis, was reported in up to 35.5% of women in the Italian cohort [28]. This estimate represents a conservative summation of clinician-documented thyroid diagnoses reported within that cohort, without assumptions regarding uniform screening or diagnostic reassessment. Taken together, these descriptive clusters outline a recurring constellation of adipose, ovarian, pelvic pain–related, and thyroid features in women with lipedema. However, given the observational nature of the data and variability in diagnostic ascertainment, these patterns should be interpreted as contextual clinical signals rather than prevalence estimates or causal associations, underscoring the need for future mechanistic and longitudinal studies.

### 3.4. Chronodependence and Hormonal Milestones

Across cohorts, the onset and progression of lipedema demonstrated consistent temporal association with key reproductive life stages, suggesting a potential modulatory role of hormonal transitions. Symptom onset was reported during puberty or adolescence in approximately 62.2% to 72.0% of cases in Swiss and German cohorts [25,30] a pattern that is further supported by patient-reported survey data indicating puberty as a frequent temporal trigger for disease onset [13]. This temporal proximity to menarche has led authors to propose that the physiological surge of sex steroids during puberty may interact with underlying biological susceptibility. In this context, the expansion of gluteofemoral adipose tissue, a normal secondary sexual characteristic, appears to become dysregulated in a subset of individuals [26].

Disease exacerbation was also frequently reported during later hormonal transitions. Approximately 53.0% of participants described worsening of symptoms during pregnancy, a period characterized by markedly elevated circulating estrogen and progesterone levels [28]. In addition, 67.9% reported symptom progression during the menopausal transition. Symptom deterioration during menopause has been discussed in relation to altered tissue level hormone signaling rather than absolute circulating hormone concentrations. Specifically, the proposed model of estrogen receptor imbalance suggests that reduced systemic estrogen signaling through *ERα* may permit increased influence of local intracrine estrogen production mediated by *ERβ* [33].

Exogenous hormonal exposure was also reported as a potential modifier of symptom expression. In the Italian cohort, 44.4% of participants reported symptom changes associated with hormonal contraceptive use, most commonly described as worsening of pain or tissue sensitivity [28,35]. While the type, dose, and duration of hormonal formulations were not systematically characterized, these observations raise the possibility of tissue specific sensitivity to exogenous hormones, without implying causality.

### 3.5. Shared Molecular and Genetic Mechanisms: A Hypothesis-Generating Perspective

Several included studies reported molecular and genetic findings that have been proposed as relevant to lipedema pathophysiology. Genetic analyses identified loss of function variants involving the *AKR1C1 gene*, which encodes *aldo keto reductase family 1 member C1*, an enzyme involved in progesterone inactivation [18]. Impaired progesterone metabolism has been discussed in relation to mechanisms described in endometriosis and adenomyosis, where progesterone resistance contributes to altered tissue remodeling and inflammatory signaling [23]. Additional associations involving *AKR1C2* suggest potential alterations in androgen and neurosteroid metabolism, although functional implications remain incompletely characterized [43].

At the tissue level, analyses of subcutaneous adipose samples demonstrated upregulation of aromatase (*CYP19A1*), the rate-limiting enzyme responsible for conversion of androgens to estrogens [21]. These findings indicate the capacity for enhanced local estrogen synthesis independent of ovarian sources. This intracrine environment was accompanied by altered estrogen receptor expression, characterized by reduced *ERα* signaling and relative predominance of *ERβ* [20]. While *ERα* is generally associated with metabolic homeostasis in adipose tissue, increased *ERβ* activity has been linked to pro inflammatory cytokine expression, including *TNF α* and *IL 6*, and to fibrotic remodeling.

Furthermore, recent multi-omics studies identified metabolic signatures distinguishing lipedema from obesity, including alterations in sphingolipid metabolism and suppression of selected immune markers [31]. These molecular profiles support the concept of lipedema adipose tissue as a biologically active and hormonally responsive compartment.

Collectively, these molecular and genetic findings describe mechanistic features that overlap conceptually with pathways reported in steroid dependent gynecological diseases. However, the available evidence remains indirect and heterogeneous, and does not establish shared etiology or causal relationships. Further experimental and longitudinal studies are required to determine the extent to which these pathways contribute to disease development, symptom expression, and clinical heterogeneity in lipedema.

## 4. Discussion

### 4.1. Synthesis of Evidence: A Hypothesis-Generating Framework Integrating Endocrine and Gynecologic Dimensions

**Figure 5:**
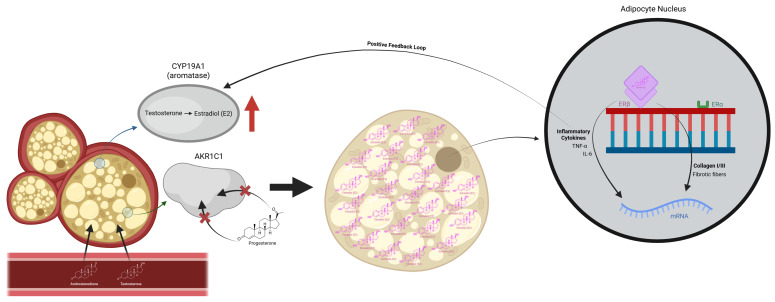
Proposed endocrine and intracrine signaling mechanisms in lipedema adipose tissue. Schematic representation summarizing hypothesized alterations in steroid metabolism and nuclear signaling in lipedema, including increased aromatase (*CYP19A1*) activity, putative alterations in progesterone metabolism associated with *AKR1C1* dysfunction, enhanced local estradiol production, and estrogen receptor imbalance with relative predominance of *ERβ* over *ERα*. These pathways are depicted in relation to inflammatory cytokine signaling and extracellular matrix remodeling. The figure represents a conceptual synthesis derived from the literature and does not imply direct causal relationships. Legend: CYP19A1—Cytochrome P450 family 19 subfamily A member 1 (aromatase), AKR1C1—Aldo-keto reductase family 1 member C1, E2—Estradiol, ERα—Estrogen receptor alpha, ERβ—Estrogen receptor beta, TNF-α—Tumor necrosis factor alpha, IL-6—Interleukin 6, mRNA—Messenger ribonucleic acid.

This scoping review consolidates emerging evidence to outline a conceptual framework that may expand current understandings of lipedema pathophysiology. Figure 5 provides a schematic representation of the proposed endocrine and intracrine mechanisms underlying the conceptual framework discussed in this section.

Conceptual model (hypothesis-generating). Schematic illustration summarizing hypothesized alterations in steroid metabolism and nuclear signaling in lipedema, including increased aromatase (*CYP19A1*) activity, impaired progesterone metabolism associated with *AKR1C1* dysfunction, enhanced local estradiol production, and estrogen receptor imbalance with relative predominance of *ERβ* over *ERα*. These pathways are depicted in relation to inflammatory cytokine signaling and extracellular matrix remodeling. The figure represents a conceptual synthesis derived from the literature and does not imply direct causal relationships.

The integration of genetic, enzymatic, and epidemiological observations suggests that mechanisms beyond lymphovascular dysfunction or adipose deposition alone may be relevant to lipedema pathophysiology. Several included studies reported disturbances in steroid-metabolizing enzymes, including *AKR1C1*, along with increased local aromatase expression, findings interpreted as consistent with enhanced intracrine estrogen activity and altered progesterone signaling [18,21]. These molecular features overlap conceptually with patterns described in estrogen-dependent gynecological conditions and have prompted hypotheses that lipedema adipose tissue may exhibit endocrine-responsive behavior with partial autonomy from systemic hormonal regulation.

Importantly, this conceptual overlap does not imply equivalence between lipedema and endometriosis, nor does the available evidence allow estimation of the true prevalence of endometriosis among women with lipedema or inference of shared etiology. Rather, the observed convergence is based on symptom clusters, reproductive life-stage associations, and indirect molecular parallels derived from heterogeneous observational and experimental studies. Within these constraints, lipedema adipose tissue may be viewed as a hormonally responsive microenvironment capable of sustaining localized inflammation and fibrotic remodeling, a biology that may plausibly contribute to the clinical resistance of lipedema-associated adipose tissue to caloric restriction. These considerations support the use of integrative metabolic and endocrine frameworks to contextualize disease heterogeneity, while underscoring the need for mechanistic and longitudinal studies to validate these proposed associations.

### 4.2. The Clinical Discordance: Unmasking the Gynecological Symptom Cluster

A notable finding of this review is the discordance between the relatively low prevalence of surgically confirmed endometriosis and the substantial burden of pelvic and menstrual symptoms reported in lipedema cohorts. While confirmed endometriosis was reported in approximately 4.2% of participants, likely reflecting underascertainment in the absence of routine laparoscopic evaluation, functional pelvic complaints were considerably more prevalent. Across cohorts, pelvic pain–related symptoms were reported in approximately 32.1% of participants, ovulatory and menstrual dysfunction (originally classified by the primary authors as ‘inflammatory ovarian dysfunction’) in 76.0%, and severe menstrual pain in 43.0%, with additional structural findings including ovarian cysts and uterine fibroids [25,28,36].

Importantly, this apparent discordance should be interpreted in light of well-recognized diagnostic constraints. Endometriosis diagnosis relies on laparoscopic visualization and histopathological confirmation, procedures that are not routinely applied in large observational cohorts or in women whose symptoms are attributed to non-gynecologic causes. As a result, studies without systematic surgical assessment are expected to underestimate true disease prevalence. In this context, dysmenorrhea and chronic pelvic pain function as sensitive but non-specific clinical markers of possible underlying pelvic pathology, rather than definitive indicators of endometriosis.

This pattern suggests that gynecological comorbidity in lipedema may be underrecognized, reflecting both diagnostic limitations and attribution of pelvic symptoms to non gynecological causes. Proposed explanatory mechanisms draw on shared molecular features, including *ERβ* predominance, reduced *ERα* signaling, and impaired progesterone responsiveness, profiles also reported in endometriotic tissue [34]. These signaling patterns have been associated with macrophage recruitment, fibrotic remodeling, and local inflammatory amplification, which may contribute to overlapping symptom expression across tissues.

While it would be premature to classify lipedema as a manifestation of systemic endometriosis, the convergence of symptom patterns raises the possibility that a subset of individuals may exhibit overlapping endocrine and inflammatory pathway activation across pelvic and subcutaneous compartments. Recognition of this overlap may help bridge existing separation between dermatological, vascular, and gynecological care pathways and encourage more integrated clinical evaluation. Further research is required to clarify prevalence, mechanisms, and clinical relevance.

### 4.3. Future Research Directions Arising from Endocrine and Metabolic Signaling

The mechanistic patterns summarized in this review raise important questions regarding how endocrine and metabolic pathways may be explored in future lipedema research. Although current management strategies focus primarily on symptomatic relief through conservative measures and surgical approaches, the molecular findings discussed suggest that disease relevant biological processes may contribute to pain, inflammation, fibrosis, and tissue dysfunction.

Evidence from estrogen-dependent gynecological disorders demonstrates that altered estrogen and progesterone signaling can influence inflammatory and stromal pathways. In endometriosis, ovulation suppressing strategies have been associated with reductions in pelvic pain, although the certainty of evidence varies and these findings are not directly transferable to lipedema [44]. In this context, such observations serve as a conceptual reference rather than therapeutic guidance, identifying endocrine signaling as a biological axis warranting further investigation.

In parallel, interest has emerged regarding pathways related to tissue fluid regulation and metabolic signaling based on observations from gynecological and metabolic research. Anti mineralocorticoid and incretin related pathways have been proposed as biologically relevant to adipose tissue function. However, in the context of lipedema, these hypotheses remain exploratory and should be regarded as priorities for experimental and early phase clinical research aimed at clarifying underlying mechanisms, rather than as indications for clinical intervention.

### 4.4. Implications for Clinical Practice

The findings of this review support consideration of a more integrated clinical approach to lipedema, extending beyond lymphovascular assessment to include metabolic, endocrine, and gynecological perspectives. Across cohorts, reproductive symptoms including infertility and pregnancy related complications were frequently reported, indicating that reproductive health concerns may represent an important dimension of the lipedema phenotype [36].

Symptom onset and progression frequently coincide with reproductive life stage transitions, including puberty, pregnancy, and menopause. In this context, awareness of gynecological comorbidities such as endometriosis or polycystic ovary syndrome may refine clinical suspicion in symptomatic individuals, without implying routine screening or diagnostic equivalence.

Regarding hormonal management, observational data consistently identify hormonal exposures as clinically relevant symptom modifiers. In a large Italian cohort, 44.4% of participants described symptom changes temporally associated with hormonal contraceptive use, most frequently reporting worsening of pain, edema, or tissue sensitivity [28]. Similarly, high rates of menstrual and ovulatory disturbances were documented in the Spanish cohort, occurring in the context of prevalent exposure to combined oral contraceptives [36]. These observations converge on a recurrent pattern of symptom aggravation potentially associated with combined estrogen–progestin formulations, particularly those containing ethinyl estradiol, rather than progestin exposure alone. This distinction mirrors paradigms in endometriosis, where estrogen suppression or progestin-dominant strategies are standard. Consequently, these findings support a hypothesis-generating rationale for exploring estrogen-free, progestin-based approaches in lipedema (e.g., drospirenone or gestrinone) [34], while noting that current evidence remains insufficient for formal guidelines. Isolated case reports of estrogen-related symptom modulation exist but remain anecdotal [26].

### 4.5. Limitations of the Evidence Base

Several limitations of the available evidence should be acknowledged. Diagnostic criteria for lipedema varied across studies and were frequently based on specialist clinical assessment rather than standardized consensus definitions. Most gynecological outcomes relied on self-report or medical record abstraction, with limited use of systematic gynecological evaluation. As endometriosis is defined surgically and histopathologically, cohorts without routine laparoscopic assessment are likely to underestimate its confirmed prevalence. In addition, many studies were conducted in specialized referral centers, introducing selection bias and limiting generalizability to broader populations. Molecular and genetic investigations were typically small and cross-sectional, restricting causal inference. Finally, substantial heterogeneity in outcome definitions limited comparability across studies and increased the likelihood of residual confounding.

## 5. Conclusions

This scoping review systematically mapped the evidence linking lipedema to gynecologic diseases. While surgically confirmed endometriosis remains infrequent, functional gynecologic symptoms such as pelvic pain and menstrual disturbances, are highly prevalent. This disparity between high symptom burden and low confirmed diagnosis suggests a systemic gap in gynecologic screening for these patients.

Symptom onset and progression consistently cluster around hormonal milestones: puberty, pregnancy, and menopause. These findings support a conceptual model where lipedema acts as a component of a broader gynecologic–endocrine constellation, rather than an isolated adipose disorder. This framework highlights convergent, hormone-sensitive pathways across tissues without implying shared etiology.

Future investigations must prioritize prospective designs with standardized gynecologic assessment and molecular profiling. Such research is essential to validate these associations, refine phenotypic classification, and advance integrated women’s health approaches.

## Figures and Tables

**Table 1:** Search Strategy.

Databases	Search Strategy	N°
ARXIV	Sorry, your query for all: LIPEDEMA produced no results.	0
BVS	(mh:(lipedema)) OR (ti:(lipedema * OR lipolinfedema OR lipoedema * OR lipolymphedema * OR lipo-lymphoedème OR lipolymphoedème)) OR (ab:(lipedema * OR lipolinfedema OR lipoedema * OR lipolymphedema * OR lipo-lymphoedème OR lipolymphoedème))	29
Dimensions	lipedema OR lipoedema OR lipolymphedema	500
EBSCO HOST	TI lipedema * OR lipoedema * OR lipolymphedema * OR AB lipedema * OR lipoedema * OR lipolymphedema * OR SU lipedema * OR lipoedema * OR lipolymphedema * OR TX lipedema * OR lipoedema * OR lipolymphedema *	903
EMBASE	lipedema/exp OR lipoedema * OR lipolymphedema *	1253
LA REFERENCE	Buscar: Lipedema * OR Lipoedema * OR Lipolymphedema *	12
Porta de Periódicos da Capes—integrated search	lipedema OR lipoedema OR lipolymphedema	41
PSYCINFO	Any Field: Lipedema * OR Any Field: Lipoedema * OR Any Field: Lipolymphedema *	15
PUBMED	Lipedema [mh] OR Lipedema * OR Lipoedema * OR Lipolymphedema *	701
SCIELO	Lipedema * OR Lipolinfedema OR Lipoedema * OR Lipolymphedema * OR Lipo-lymphoedème OR Lipolymphoedème	12
SCOPUS	TITLE-ABS-KEY (lipedema * OR lipoedema * OR lipolymphedema *)	1104
WOS	TS = (lipedema * OR lipoedema * OR lipolymphedema *)	677
Total		5247

Legend: mh = Medical Subject Headings (e.g., MeSH); /exp = explosion of the controlled vocabulary term; ti = title field; ab = abstract field; TI = title; AB = abstract; SU = subject terms; TX = full text or all text fields; TITLE-ABS-KEY = title, abstract, and keywords; TS = topic (title, abstract, author keywords, and Keywords Plus); Any Field = search across all available fields; OR = Boolean operator used to combine synonyms; * = truncation symbol to retrieve word variants.

**Table 2:** Differentiation between Confirmed Gynecologic Diagnoses and Symptom-Based Outcomes in Lipedema Cohorts. Note: Percentages represent weighted prevalence or ranges reported across included studies. The 76.0% value for “Menstrual Irregularities” refers to the composite “inflammatory ovarian dysfunction” cluster defined by Simarro et al. (2025) [36].

Category	Outcome of Interest	Method of Ascertainment	Reported Prevalence
Confirmed Diagnoses	Endometriosis	Surgical/Histopathological Confirmation	4.2%
	Polycystic Ovary Syndrome (PCOS)	Medical Diagnosis (Rotterdam Criteria/History)	12.6–17.1%
	Uterine Fibroids (Leiomyoma)	Imaging/Medical History	15.2%
	Thyroid Dysfunction	Laboratory/Medical History	35.5–59.0%
Symptom-Based Results	Menstrual Pain (Dysmenorrhea)	Self-Reported (Questionnaire)	43.0%
	Pelvic Pain Cluster	Self-Reported (Cycle-related pain)	32.1%
	Menstrual Irregularities	Self-Reported/Clinical Interview	32.5–76.0%
	Infertility	Self-Reported History	18.0%

## Data Availability

No new data were created or analyzed in this study.

## Abbreviations

AKR1C1	Aldo-Keto Reductase Family 1 Member C1
AKR1C2	Aldo-Keto Reductase Family 1 Member C2
ASCs	Adipose-Derived Stromal/Stem Cells
AT	Adipose Tissue
BMI	Body Mass Index
CAV1	Caveolin-1
CNN	Convolutional Neural Network
CYP19A1	Aromatase (Cytochrome P450 Family 19 Subfamily A Member 1)
EC	Endothelial Cells
ECM	Extracellular Matrix
ERα	Estrogen Receptor Alpha
ERβ	Estrogen Receptor Beta
ESR1	Estrogen Receptor 1 Gene
ESR2	Estrogen Receptor 2 Gene
IL-6	Interleukin 6
IL-8	Interleukin 8
IJMS	International Journal of Molecular Sciences
JBI	Joanna Briggs Institute
LPS	Lipopolysaccharide
MMP14	Matrix Metalloproteinase 14
NRS	Numeric Rating Scale
OSF	Open Science Framework
PCOS	Polycystic Ovary Syndrome
PMDD	Premenstrual Dysphoric Disorder
PRESS	Peer Review of Electronic Search Strategies
PRISMA-ScR	Preferred Reporting Items for Systematic Reviews and Meta-Analyses Extension for Scoping Reviews
PROX1	Prospero Homeobox 1
QoL	Quality of Life
SAT	Subcutaneous Adipose Tissue
SVF	Stromal Vascular Fraction
TNF-α	Tumor Necrosis Factor Alpha
WES	Whole-Exome Sequencing
WHO	World Health Organization
